# Double Doping of a Low-Ionization-Energy Polythiophene with a Molybdenum Dithiolene Complex

**DOI:** 10.1021/acs.chemmater.2c01040

**Published:** 2022-06-13

**Authors:** Emmy Järsvall, Till Biskup, Yadong Zhang, Renee Kroon, Stephen Barlow, Seth R. Marder, Christian Müller

**Affiliations:** † Department of Chemistry and Chemical Engineering, Chalmers University of Technology, 41296 Göteborg, Sweden; ‡ Physical Chemistry, University of Saarland, Saarbrücken 66123, Germany; § Georgia Institute of Technology, School of Chemistry and Biochemistry and Center for Organic Photonics and Electronics, Atlanta, Georgia 30332-0400, United States; ∥ Renewable and Sustainable Energy Institute, University of Colorado Boulder, Boulder, Colorado 80303, United States; ⊥ Laboratory of Organic Electronics, Linköping University, 60174 Norrköping, Sweden; # Departments of Chemical and Biological Engineering and of Chemistry, University of Colorado Boulder, Boulder, Colorado 80303, United States

## Abstract

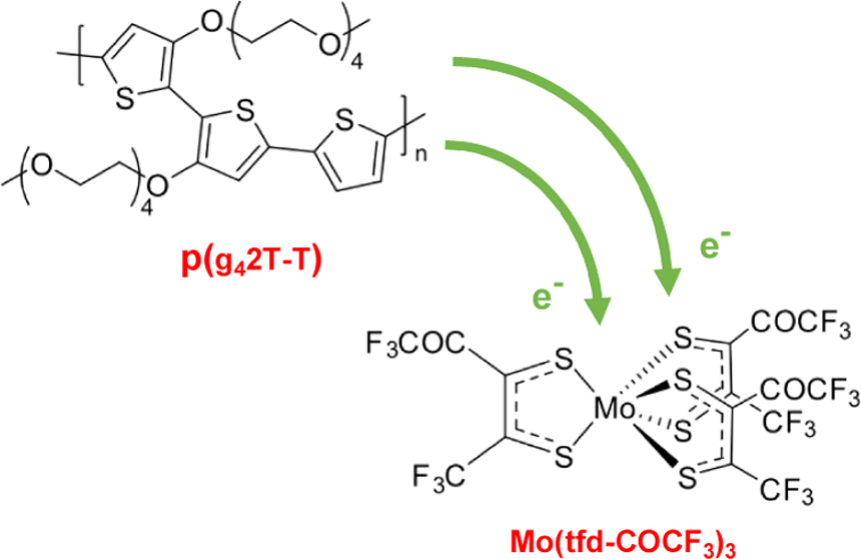

Doping of organic semiconductors is crucial for tuning the charge-carrier density of conjugated polymers. The exchange of more than one electron between a monomeric dopant and an organic semiconductor allows the polaron density to be increased relative to the number of counterions that are introduced into the host matrix. Here, a molybdenum dithiolene complex with a high electron affinity of 5.5 eV is shown to accept two electrons from a polythiophene that has a low ionization energy of 4.7 eV. Double p-doping is consistent with the ability of the monoanion salt of the molybdenum dithiolene complex to dope the polymer. The transfer of two electrons to the neutral dopant was also confirmed by electron paramagnetic resonance spectroscopy since the monoanion, but not the dianion, of the molybdenum dithiolene complex features an unpaired electron. Double doping allowed an ionization efficiency of 200% to be reached, which facilitates the design of strongly doped semiconductors while lessening any counterion-induced disruption of the nanostructure.

## Introduction

Organic semiconductors attract a great deal of attention since they enable the development of lightweight, flexible, and biocompatible electronic devices for applications from energy harvesting and storage to bioelectronics, which cannot be achieved with inorganic semiconductors alone.^1,2^ Doping is a widely used tool to tune the charge-carrier density, which allows the electrical properties of organic semiconductors to be optimized.^3−5^ The process typically involves the addition of a dopant, such as an organic molecule, a metal–organic complex, or a metallic salt, that undergoes an electron transfer with the organic semiconductor.^5^ Electron transfer readily occurs in the case of a favorable energetic offset between the two materials. In the case of p-doping, for example, it is beneficial if the dopant has an electron affinity (EA) that is larger than the ionization energy (IE) of the semiconductor, i.e., EA_dopant_ ≥ IE_semiconductor_. Here, it is important to note that the EA and IE, which are often estimated from electrochemical methods, can take on very different values once the dopant and semiconductor are mixed.^6^ Furthermore, the structural and energetic disorder inherent to many organic semiconductors leads to a broad density of states, which may facilitate some degree of electron transfer despite an unfavorable energy offset.^7,8^ Provided that each p-dopant accepts one electron from the semiconductor, i.e., the pair undergoes integer charge transfer, one polaron is created per dopant molecule, resulting in an ionization efficiency of 100%. The ionization efficiency is reduced to less than 100% in the case that only partial charge transfer occurs, if there is an unfavorable energetic offset between EA_dopant_ and IE_semiconductor_ or if the dopant molecules aggregate. The presence of excess dopants and dopant aggregates unduly disrupts the nanostructure of the semiconductor, which tends to negatively affect the electrical properties.^9−11^

We have recently reported that quinodimethane-type dopants with a sufficiently high EA_dopant_ can accept two electrons from a low-IE conjugated polymer, resulting in an ionization efficiency of 200%.^12^ This type of double doping was observed for a thienothiophene-based copolymer with tetraethylene glycol side chains, for which IE_polymer_ = 4.5 eV, doped with either 2,3,5,6-tetrafluoro-7,7,8,8-tetracyanoquinodimethane (F_4_TCNQ), for which EA_F_4_TCNQ_ = 5.2 eV, or 1,3,4,5,7,8-hexafluorotetracyanonaphthoquinodimethane (F_6_TCNNQ), for which EA_F_6_TCNNQ_ = 5.3 eV (electron affinity estimated from half-wave potential *E*_1/2_ vs ferrocene/ferrocenium, Fc/Fc^+^; EA_dopant_ = 5.1 eV + *E*_1/2_).^12^ The electron affinities of the anions of these two quinodimethane-type dopants, EA_dopant_^–^, are estimated to remain sufficiently low that EA_dopant_^–^ > IE_polymer_, allowing each dopant to accept a second electron from the polymer. Other dopants that can give rise to more than one polaron include the dimers formed by certain 19-electron organometallic sandwich compounds or by benzoimidazoline radicals, the overall reaction of which with a host results in the formation of two monomeric dopants and the release of two electrons.^7^ These dopant dimers, however, have approximately twice the molecular volume of related monomeric dopants, which reduces the benefit of double doping because it doubles the overall volume that is occupied by counterions. Likewise, multivalent radical cation salts that comprise two or four triphenylamine units can accept two or even four electrons from a conjugated polymer^13,14^ but have a close to 2–4 times larger volume compared to the corresponding monovalent radical anion salt tris(4-bromophenyl)ammoniumyl hexachloroantimonate ([TPA–Br_3_]^+^[SbCl_6_]^−^; Magic Blue). We argue that double doping is preferably achieved without increasing the size of the dopant so that fewer dopant molecules must be added to obtain a certain polaron density, which reduces the risk of dopant aggregation and lessens the impact of doping on the nanostructure of the polymer.

**Figure 1:**
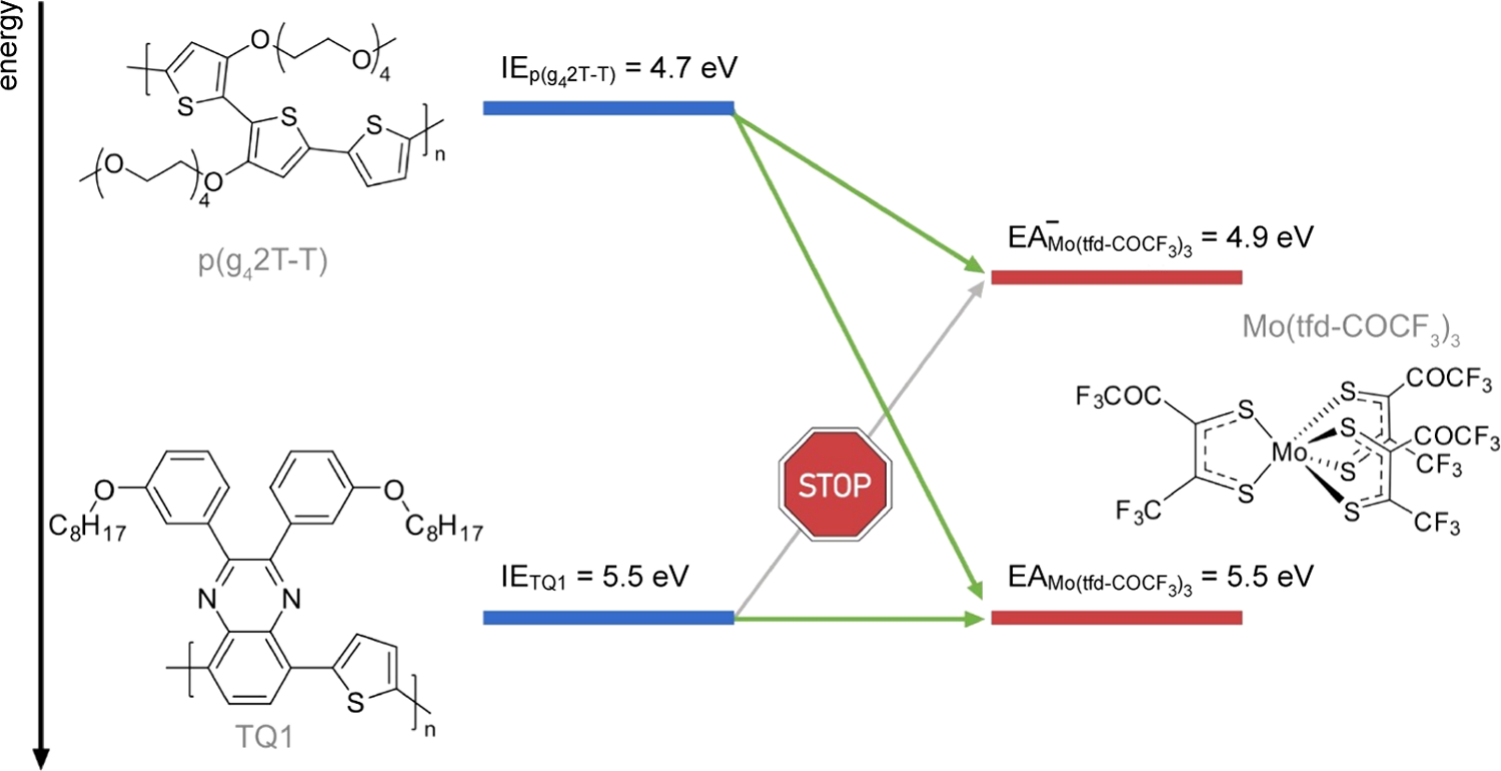
Energy diagram showing the energy levels that are relevant for charge transfer from polymers p(g_4_2T-T) and TQ1 to the p-type dopant Mo(tfd-COCF_3_)_3_.

Here we ask whether double doping is unique to quinodimethane-type monomeric dopants or whether it is a generic concept that can also be observed for other species. In particular, we investigate doping using the dithiolene complex Mo(tfd-COCF_3_)_3_,^15^ for which EA_Mo(tfd-COCF_3_)_3__ can be estimated as ca. 5.5 eV (reduction potential *E*_red_ ≈ +0.39 V vs decamethylferrocene/decamethylferrocenium, DmFc/DmFc^+^; EA_Mo(tfd-COCF_3_)_3__ = 5.1 eV + *E*_red_).^16^ We note that electrochemically based estimates of the electron affinity are approximate because solvation effects and solid-state polarization effects likely vary between molecules of different sizes and shapes. However, the value of EA_Mo(tfd-COCF_3_)_3__ = 5.5 eV is in reasonable agreement with a value of 5.6 eV measured in the solid state by inverse photoelectron spectroscopy for a related molecule, Mo(tfd)_3_, for which *E*_red_ ≈ +0.28 V vs DmFc/DmFc^+^.^17^ Mo(tfd-COCF_3_)_3_ has been used to dope a wide range of conjugated polymers including poly(3-hexylthiophene) (P3HT),^18,19^ poly[2,5-bis(3-tetradecylthiophen-2-yl)thieno[3,2-*b*]thiophene] (PBTTT),^20,21^ and a benzodithiophene-based copolymer.^9,22,23^ We chose Mo(tfd-COCF_3_)_3_ because it can exist both as an anion and dianion, meaning that it can sustain two electron transfer events, and both of these species are known to have reasonable chemical stability (indeed, the neutral dopant is obtained by chemical oxidation of the dianion).^16^ The Mo(tfd-COCF_3_)_3_ anion electron affinity, EA_Mo(tfd–COCF_3_)_3__^–^, can be estimated as ca. 4.9 eV (*E*_red_ ≈ −0.16 V vs Fc/Fc^+^),^16^ although the potential separation between subsequent redox processes in electrochemical experiments can be highly medium-dependent,^24^ meaning there will be a larger uncertainty in this estimate than in those of the electron affinity of neutral molecules. Nevertheless, if EA_Mo(tfd–COCF_3_)_3__^–^ is indeed ca. 4.9 eV, then polymers with an IE_polymer_ < 4.9 eV should be able to experience double doping when brought in contact with Mo(tfd-COCF_3_)_3_. Doping of two polymers was investigated, the polythiophene p(g_4_2T-T) with tetraethylene glycol side chains and a low IE_p(g_4_2T–T)_ = 4.7 eV (oxidation potential *E*_ox_ ≈ −0.44 V vs Fc/Fc^+^; IE_p(g_4_2T–T)_ = 5.1 eV + *E*_ox_)^25^ and the thiophene-quinoxaline copolymer TQ1,^26,27^ which features a large IE_TQ1_ = 5.5 eV (*E*_ox_ ≈ 0.37 V vs Fc/Fc^+^;^27^ see Figure 1 for chemical structures and energy levels). We here show that p(g_4_2T-T) readily undergoes double doping by Mo(tfd-COCF_3_)_3_.

## Results and Discussion

**Figure 2:**
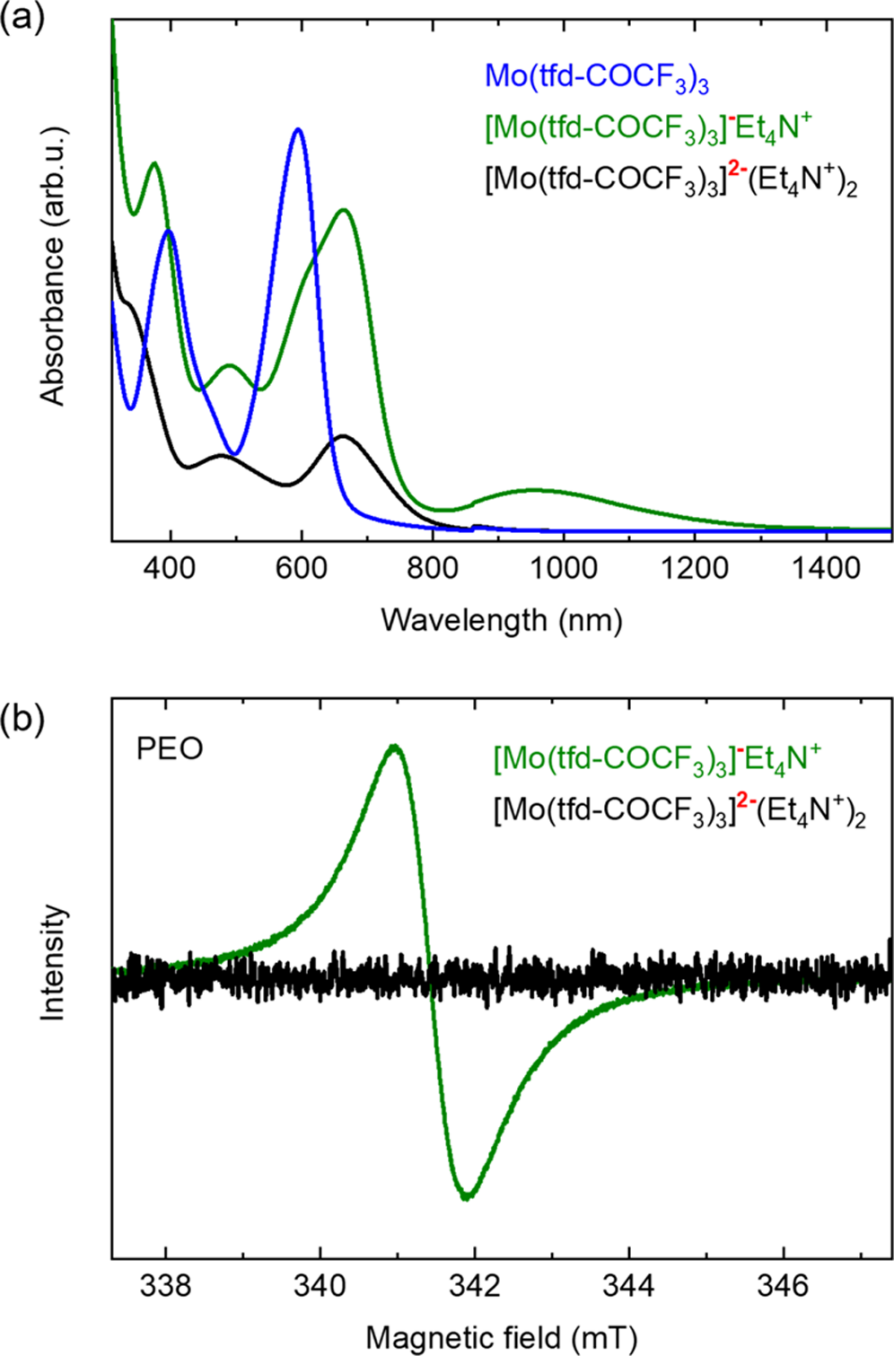
(a) UV–vis spectra of Mo(tfd-COCF_3_)_3_, [Mo(tfd-COCF_3_)_3_]^−^Et_4_N^+^, and [Mo(tfd-COCF_3_)_3_]^2–^(Et_4_N^+^)_2_ dissolved in dichloromethane (DCM). (b) EPR spectra of [Mo(tfd-COCF_3_)_3_]^−^Et_4_N^+^ and [Mo(tfd-COCF_3_)_3_]^2–^(Et_4_N^+^)_2_ dispersed in a matrix of poly(ethylene oxide) (PEO; see Figure S4 for nonsmoothed EPR spectra).

The dopant investigated here can exist as a neutral complex, Mo(tfd-COCF_3_)_3_, as well as a salt comprising either its anion or dianion. The dianion salt Mo(tfd-COCF_3_)_3_(Et_4_N)_2_ is a precursor for the synthesis of Mo(tfd-COCF_3_)_3_, as reported previously.^15,16^ Neutral Mo(tfd-COCF_3_)_3_ was prepared by oxidizing the dianion salt using NOPF_6_. The anion salt, instead, was prepared by the comproportionation of equimolar amounts of Mo(tfd-COCF_3_)_3_ and the dianion salt Mo(tfd-COCF_3_)_3_ (Et_4_N)_2_. All three species give rise to distinct ultraviolet–visible–near-infrared (UV–vis–NIR) absorbance spectra (Figure 2a). These absorptions, however, occur at wavelengths where either neat p(g_4_2T-T) and TQ1 absorb or where polaronic absorbance peaks tend to arise upon doping. Hence, it is challenging to extract information about the presence of different species from optical spectroscopy. Instead, we chose to use electron paramagnetic resonance (EPR) spectroscopy to detect the presence of different species.

In a first set of experiments, we recorded EPR spectra of the monoanion salt and the dianion salt dispersed in poly(ethylene oxide) (PEO) to mimic the solid-state environment that the two species will experience when dispersed in a p(g_4_2T-T) matrix. The UV–vis–NIR spectra of the monoanion and dianion salt dissolved in dichloromethane (DCM) or in PEO are comparable (Figure S1), which suggests that the PEO matrix does not strongly alter the electronic state of the two species. The monoanion salt gives rise to a distinct EPR signal centered at *B*_0_ = 341.4 mT, in agreement with reports for other monoanionic molybdenum tris(dithiolene) complexes,^28−30^ while the dianion salt appears spin-silent (Figures 2b and S2–S4), consistent with the sharp NMR spectra reported for [Mo(tfd-COCF_3_)_3_]^2–^,^15^ as well as with magnetic susceptibility^30^ and computational studies^31^ of other molybdenum tris(dithiolene) dianions.

**Figure 3:**
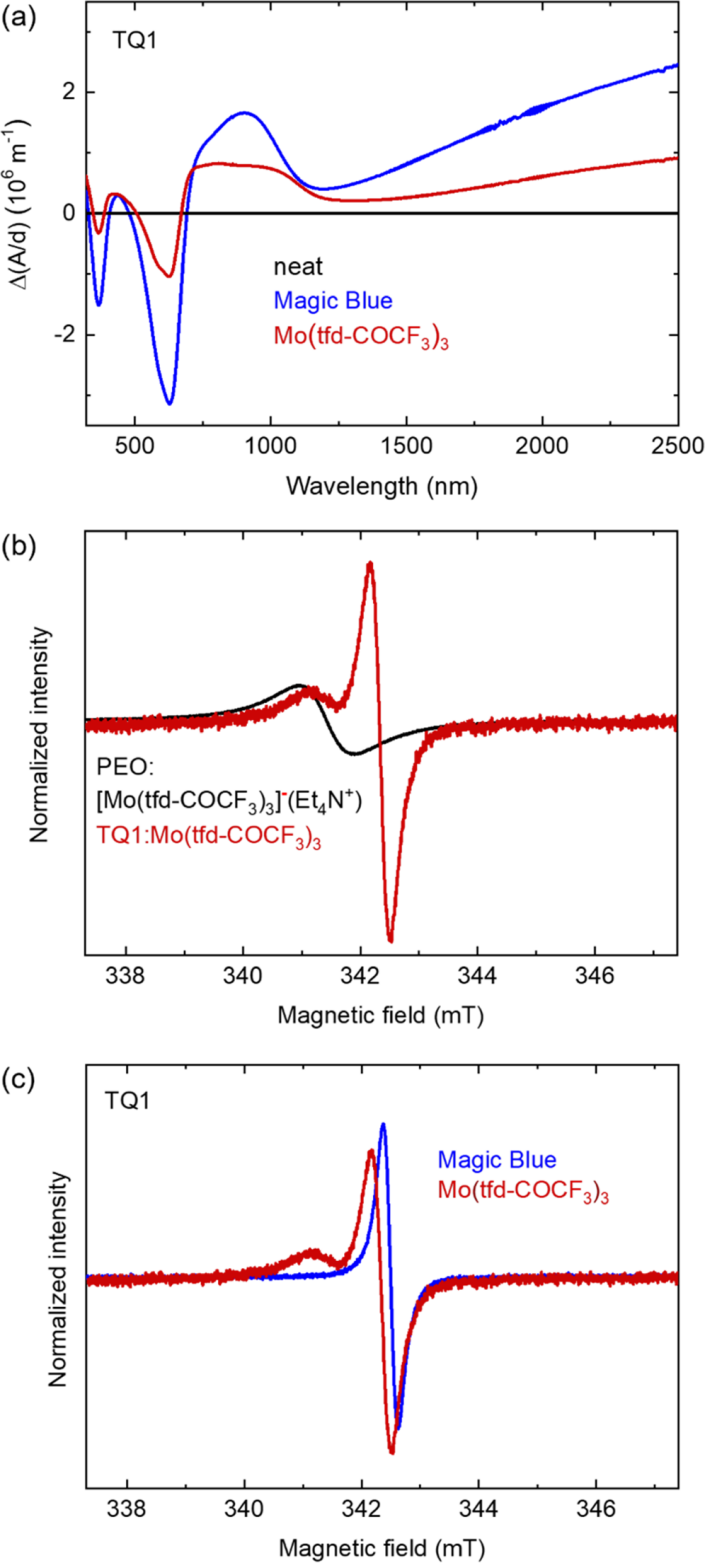
(a) UV–vis–NIR absorbance spectra displaying the difference in thickness-normalized absorbance Δ(*A*/*d*) between the spectra of neat TQ1 and TQ1 doped with Magic Blue or Mo(tfd-COCF_3_)_3_. (b) EPR spectra of [Mo(tfd-COCF_3_)_3_]^−^Et_4_N^+^ dispersed in a PEO matrix and TQ1 doped with Mo(tfd-COCF_3_)_3_. The spectra have been normalized to the maximum in the field range 338–341.5 mT. (c) EPR spectra of TQ1 doped with Magic Blue or Mo(tfd-COCF_3_)_3_. The spectra have been normalized to the same amplitude.

We next studied doped TQ1, which has a high IE_TQ1_ = 5.5 eV that is comparable to EA_Mo(tfd-COCF_3_)_3__. Judging by the ionization energy of TQ1, the polymer can be expected to undergo some electron transfer with neutral Mo(tfd-COCF_3_)_3_, but the energy levels of TQ1 and [Mo(tfd-COCF_3_)_3_]^−^ should not allow for a second charge transfer event since IE_TQ1_ ≫ EA_Mo(tfd–COCF_3_)_3__^–^. UV–vis–NIR spectra of TQ1 sequentially doped with Mo(tfd-COCF_3_)_3_ reveal clear polaronic absorption bands confirming that electron transfer has indeed occurred (Figure 3a). The EPR spectra for TQ1 doped with Mo(tfd-COCF_3_)_3_ consist of two overlapping signals, one at a magnetic field of 342.2 mT and one at a lower magnetic field. We assign the signal at a lower magnetic field to the monoanion of Mo(tfd-COCF_3_)_3_ since it appears at the same position as the EPR signal observed for [Mo(tfd-COCF_3_)_3_]^−^Et_4_N^+^ (Figures 3b, S5, and S6). This observation suggests that doping of TQ1 with Mo(tfd-COCF_3_)_3_ primarily occurs through a single electron transfer event per dopant, resulting in Mo(tfd-COCF_3_)_3_ anions that can be detected with EPR spectroscopy.

To confirm the plausibility of assigning the EPR signal at 342.2 mT to the polymer polaron, we also recorded EPR spectra of TQ1 doped with Magic Blue, which is a stronger oxidant than Mo(tfd-COCF_3_)_3_ and in which the radical cation portion can accept an electron from TQ1 because neutral tris(4-bromophenyl)amine (TPA–Br_3_) has a very high IE_TPA–Br_3__ ≈ 5.7 eV (*E*_1/2_ = +0.7 V vs DmFc/DmFc^+^),^32^ i.e., its radical cation [TPA–Br_3_]^+^ can be reduced by TQ1 since 5.7 eV > IE_TQ1_. Doping of TQ1 with Magic Blue is confirmed by the appearance of a strong polaron absorption band at 900 nm in recorded UV–vis spectra (Figure 3a).^33^ The tris(4-bromophenyl)ammoniumyl cation [TPA–Br_3_]^+^ becomes neutral as it receives an electron from the polymer during the doping process, and hence ends up spin-silent. Consequently, the EPR spectrum of doped TQ1 exclusively features a polaron signal. The similarity of the signal at ca. 342.2 mT seen for Mo(tfd-COCF_3_)_3_-doped TQ1 to the EPR spectrum of TQ1 doped with Magic Blue confirms that this signal arises from polarons on TQ1 (Figures 3c, S7, and S8).

**Figure 4:**
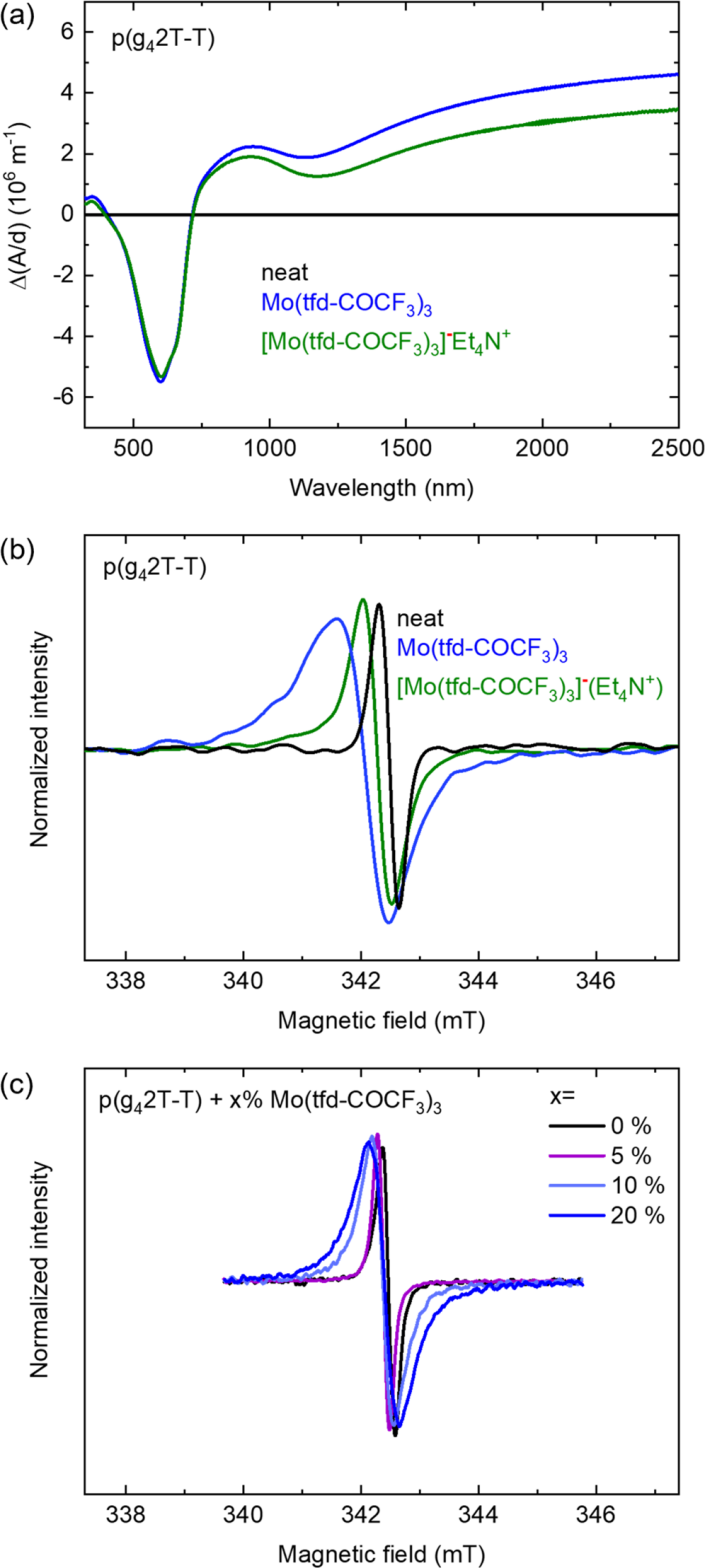
(a) UV–vis–NIR absorbance spectra displaying the difference in thickness-normalized absorbance Δ(*A*/*d*) between the spectra of neat p(g_4_2T-T) and p(g_4_2T-T) sequentially doped with Mo(tfd-COCF_3_)_3_ or [Mo(tfd-COCF_3_)_3_]^−^Et_4_N^+^. (b) EPR spectra of neat p(g_4_2T-T) and p(g_4_2T-T) sequentially doped with Mo(tfd-COCF_3_)_3_ or [Mo(tfd-COCF_3_)_3_]^−^Et_4_N^+^. (c) EPR spectra of co-processed p(g_4_2T-T):Mo(tfd-COCF_3_)_3_ films (see Figures S9 and S11 for nonsmoothed EPR spectra).

In a further set of experiments, we studied sequential doping of p(g_4_2T-T) with neutral Mo(tfd-COCF_3_)_3_ as well as its anion salt, i.e., the two species were allowed to ingress from a dopant solution into a solid film of the polymer (see the Experimental Section for details). UV–vis–NIR spectroscopy reveals stronger polaron absorption bands at 900 nm and in the near-infrared region for p(g_4_2T-T) doped with the neutral complex, which indicates a higher degree of doping with Mo(tfd-COCF_3_)_3_ compared to doping with the monoanion salt (Figure 4a). Moreover, the fact that we can dope p(g_4_2T-T) with the monoanion suggests that double doping is possible for this system. Doping of p(g_4_2T-T) with Mo(tfd-COCF_3_)_3_ gives rise to an electrical conductivity of σ = (19.6 ± 0.6) S cm^–1^, whereas doping with the monoanion salt gives a value of σ = (11.9 ± 0.3) S cm^–1^. The higher electrical conductivity for p(g_4_2T-T) doped with the neutral complex is also an indication that we have a higher degree of doping, i.e., a larger number of charge carriers in this sample compared to p(g_4_2T-T) doped with the monoanion salt. EPR spectra of neat p(g_4_2T-T) as well as the polymer doped with either neutral Mo(tfd-COCF_3_)_3_ or the anion salt all reveal a polaron signal at *g* = 342.2 mT (Figures 4b and S9). The signal in the case of the neat p(g_4_2T-T) sample likely arises from adventitious oxygen doping of the polymer. In the case of doping with the neutral complex, the recorded EPR spectrum features a broad shoulder at a lower magnetic field around 341 mT, which we explain with the presence of monoanions, i.e., the EPR spectrum is a superposition of the EPR signal of the polaron on the polymer and the EPR signal of the monoanion. The EPR spectrum of p(g_4_2T-T) sequentially doped with the monoanion features a signal at a similar field as the neat, oxygen-doped polymer, and the absence of a broad shoulder around 341 mT suggests that no major fraction of monoanions is present. Since UV–vis–NIR spectroscopy indicates that the polymer was doped, i.e., polarons were generated (see Figure 4a), the majority of monoanions that have entered the polymer film must have accepted an electron from p(g_4_2T-T) and thus became spin-silent dianions.

Finally, we studied a series of p(g_4_2T-T) thin films that were prepared by co-processing the polymer with different amounts of the neutral Mo-complex. The conductivity increases with the amount of dopant (Table 1) and the polaron absorbance increases (see UV–vis–NIR spectra in Figure S10), which is consistent with an increasing polaron concentration. For all co-processed samples, the EPR signal is centered at 342.2 mT, i.e., the position assigned to polarons, and we do not observe any broad shoulder at lower magnetic fields (Figures 4c and S11). However, we instead observe a symmetrical broadening with increasing dopant concentration. We interpret the lack of the low-field shoulder as an indication that no significant amount of monoanions is present and hence the EPR signal solely originates from the polarons. The observed symmetrical broadening of the EPR signal with increasing dopant concentration could be attributed to a spin–spin interaction between nearby polarons.^34^ Another explanation for the observed symmetrical broadening could be a lower degree of interchain charge hopping with increasing dopant concentrations resulting in the transition from Lorentzian to Gaussian absorption features.^35^

**Table 1:** Parameters Quantifying the Extent of Doping and the Resulting Conductivity and Mobility for p(g_4_2T-T) Co-Processed with Different Amounts of Mo(tfd-COCF_3_)_3_; Amount of Dopant in mol %, Calculated Relative to the Molar Mass of the Repeat Unit of p(g_4_2T-T), Oxidation Level *O*_ox_, i.e., Number of Polarons Per Polymer Thiophene Ring in Percent, Ionization Efficiency η_ion_, i.e., Number of Polarons Per Added Dopant Molecules in Percent, Number of Polarons *N*_p_, Conductivity σ and Mobility μ = σ*eN*_p_, where *e* is the Elementary Charge

mol % dopant	*O*_ox_ (%)	η_ion_ (%)	*N*_p_ (10^26^ m^–3^)	σ (S cm^–1^)	μ (cm^2^ V^–1^ s^–1^)
5	3.4 ± 0.2	195 ± 3	0.94 ± 0.02	0.05 ± 0.01	0.003 ± 0.001
10	7.4	200^a^	2.0	1.1 ± 0.1	0.03 ± 0.01
20	13.1 ± 0.2	158 ± 2.3	3.6 ± 0.1	15 ± 1	0.26 ± 0.04

a For 10 mol % dopant, the calculated value of η_ion_ was larger than physically possible and therefore was set to 200%.

We used the UV–vis–NIR spectra of the co-processed samples to estimate the number of polarons *N*_p_ (Table 1). To estimate *N*_p_ we compared the thickness-normalized difference in absorbance Δ(*A*_800_/*d*) between doped samples and the neat polymer at 800 nm, where a pronounced sub-bandgap polaronic absorption peak is situated, with the molar attenuation coefficient of the polaronic absorption obtained from electrochemically doped P3HT at 800 nm, ε_800_ = (4.1 ± 0.2) × 10^3^ m^2^ mol^–1^.^19^ The Beer–Lambert law Δ(*A*_800_/*d*) = ε_800_ × *N*_p_, where *d* is the film thickness, was used to estimate *N*_p_. We then calculated the oxidation level according to *O*_ox_ = *N*_p_/*N*_thiophene_ and ionization efficiency according to η_ion_ = *N*_p_/*N*_Mo(tfd–COCF_3_)_3__, where *N*_Mo(tfd–COCF_3_)_3__ is the number of molybdenum dithiolene complexes (Table 1). We obtain an ionization efficiency of more than 100% for all studied samples, indicating that double doping occurs even for the samples with a higher doping concentration. For 5 and 10 mol % dopants, we estimate that the ionization efficiency is close to 200%, meaning that each Mo(tfd-COCF_3_)_3_ complex accepts two electrons from p(g_4_2T-T). A high degree of double doping explains why we do not observe any clear feature of the monoanion in the EPR spectra of p(g_4_2T-T) co-processed with Mo(tfd-COCF_3_)_3_ (Figure 4c). Most of the monoanions that form undergo a second electron transfer event and thus become dianions, which are spin-silent.

## Conclusions

We conclude that the molybdenum dithiolene complex Mo(tfd-COCF^_3_^)_3_ can accept two electrons from the conjugated polymer p(g_4_2T-T). The dopant monoanions could be detected with EPR spectroscopy because their signal appears at a lower magnetic field than the signal from the polymer polaron. The absence of a clear monoanion signal in EPR spectra of the polymer co-processed with Mo(tfd-COCF_3_)_3_ suggests that only dianions are present, i.e., the dopant underwent two electron transfer events with the polymer. The viability of double doping was confirmed by sequential doping of polymer films with the monoanion salt [Mo(tfd-COCF_3_)_3_]^−^Et_4_N^+^, which gave rise to a conductivity of σ = (11.9 ± 0.3) S cm^–1^ and EPR spectra that did not feature any signal from anions, indicating that each anion had been converted into a spin-silent dianion by accepting one electron from the polymer. Analysis of the UV–vis–NIR spectra of co-processed samples indicated an ionization efficiency of 200% for use of up to 10 mol % Mo(tfd-COCF_3_)_3_. It can be anticipated that double doping of polymers with suitable monomeric dopants may allow us to achieve high charge-carrier densities while reducing the number of counterions and hence their collective impact on the nanostructure of semiconductor films.

## Experimental Section

### Materials

TQ1 (weight- and number-average molecular weights, *M*_n_ = 76 kg mol^–1^ and PDI = 2.6), p(g_4_2T-T) (*M*_n_ = 24 kg mol^–1^, PDI = 3.3), Mo(tfd-COCF_3_)_3_, and [Mo(tfd-COCF_3_)_3_]^−^Et_4_N^+^ and [Mo(tfd-COCF_3_)_3_]^2–^(Et_4_N^+^)_2_ were prepared according to previously reported procedures.^15,27,36^ Poly(ethylene oxide) (PEO, *M*_w_ = 200 kg mol^–1^), [TPA–Br_3_]^+^[SbCl_6_]^−^ (Magic Blue), dichloromethane (DCM, purity > 99.9%), and anhydrous acetonitrile (AcN, purity > 99.8%) were purchased from Sigma-Aldrich. Chlorobenzene (CB, purity > 99%) and chloroform (CHCl_3_, purity > 99.8%) were obtained from Fisher Scientific. All commercial solvents and the Magic Blue dopant were used as received and without further purification.

### Sample Preparation

Polymers were dissolved with a concentration of 10 g L^–1^ (TQ1 in CB and p(g_4_2T-T) in CHCl_3_) and spin-cast onto poly(ethylene terephthalate) (PET) films, yielding a film thickness of 50–100 nm. The polymer films were sequentially doped with Magic Blue dissolved in AcN/CHCl_3_ (3:1, v/v; 0.45 g L^–1^), Mo(tfd-COCF_3_)_3_ in AcN/CHCl_3_ (3:1, v/v; 2.5 g L^–1^), and [Mo(tfd-COCF_3_)_3_]^−^Et_4_N^+^ in AcN/DCM (3:2, v/v; 2.5 g L^–1^). The dopant solutions were added on top of the films for 30 s, spun off, and finally the films were rinsed with anhydrous AcN to remove any excess dopant on top of the film. To prepare co-processed p(g_4_2T-T)/Mo(tfd-COCF_3_)_3_ samples, p(g_4_2T-T) and Mo(tfd-COCF_3_)_3_ were dissolved in AcN/CHCl_3_ (1:1, v/v; 15 and 5 g L^–1^). Appropriate volumes of AcN/CHCl_3_ (1:1, v/v) were then added to the p(g_4_2T-T) solutions before the addition of the Mo(tfd-COCF_3_)_3_ solution to maintain a polymer concentration of 5–7.5 g L^–1^ in each polymer/dopant solution while varying the concentration of Mo(tfd-COCF_3_)_3_. The co-processed polymer/dopant solutions were spin-cast onto PET films, yielding a film thickness of 80–200 nm. The calculations of molar percentage were based on the molar mass of the polymer repeat unit and the molar mass of the dopant. Mo(tfd-COCF_3_)_3_, [Mo(tfd-COCF_3_)_3_]^−^Et_4_N^+^, and [Mo(tfd-COCF_3_)_3_]^2–^(Et_4_N^+^)_2_ were dissolved together with PEO in DCM (4 and 16 g L^–1^), and the solutions were then spin-cast onto PET films.

### UV–vis–NIR Absorption Spectroscopy

Measurements of liquid and solid samples were performed with a PerkinElmer Lambda 1050 spectrometer. Mo(tfd-COCF_3_)_3_, [Mo(tfd-COCF_3_)_3_]^−^Et_4_N^+^, and [Mo(tfd-COCF_3_)_3_]^2–^(Et_4_N^+^)_2_ solutions for UV–vis–NIR absorption measurements were prepared at a concentration of 1 g L^–1^ in DCM.

### Electron Paramagnetic Resonance

The polymer films coated on PET films were cut to a size of 25 mm × 3 mm and then sealed under nitrogen in a quartz EPR tube. EPR spectra were recorded at room temperature using a Magnettech MS5000 spectrometer (Freiberg Instruments, now Bruker Biospin). All spectra were corrected for the same microwave frequency (9.6 GHz). Data processing and analysis have been performed using the cwepr Python package.^37,38^

### Electrical Characterization

The electrical resistivity was measured with a four-point probe setup from Jandel Engineering (cylindrical probe head, RM3000) using collinear tungsten carbide electrodes with an equidistant spacing of 1 mm that were held down with a constant weight of 60 g. The electrical conductivity σ was then calculated according to σ = ((*V*/*I*)*kt*)^−1^, where *V* is the voltage, *I* is the current, *k* = 4.53 is a geometrical correction factor, and *t* is the thickness.

## Supporting Information Available

UV–vis–NIR spectra as well as angular-dependent and nonsmoothed EPR spectra (PDF)

## Notes

EPR raw data and analysis “recipes” used to analyze the EPR data are available online via Zenodo (DOI: 10.5281/zenodo.6592331).
