# Supplementary material for: Double Doping of a Low-Ionization-Energy Polythiophene with a Molybdenum Dithiolene Complex
Source: Chem Mater. 2022 Jun 13;34(12):5673–9. doi: 10.1021/acs.chemmater.2c01040 (PMC9245179; doi:10.1021/acs.chemmater.2c01040)
Supplement: Supplementary file 1 — cm2c01040_si_001.pdf [file cm2c01040_si_001.pdf]

Supporting information

Double doping of a low-ionization energy polythiophene with a molybdenum dithiolene complex

Emmy Järsvall,[a] Till Biskup,[b] Yadong Zhang,[c,d] Renee Kroon,[a,e] Stephen Barlow,[c,d] Seth R. Marder,[c,d,f] Christian Müller[a]*

[a]Department of Chemistry and Chemical Engineering, Chalmers University of Technology, 41296 Göteborg, Sweden

[b]Physical Chemistry, University of Saarland, Saarbrücken 66123, Germany

[c]Georgia Institute of Technology, School of Chemistry and Biochemistry and Center for Organic Photonics and Electronics, Atlanta, Georgia 30332-0400, United States

[d]Renewable and Sustainable Energy Institute, University of Colorado Boulder, Boulder, CO 80303, United States

[e]Laboratory of Organic Electronics, Linköping University, 60174 Norrköping, Sweden

[f]Departments of Chemical and Biological Engineering and of Chemistry, University of Colorado Boulder, Boulder, CO 80303, United States

*e-mail: christian.muller@chalmers.se

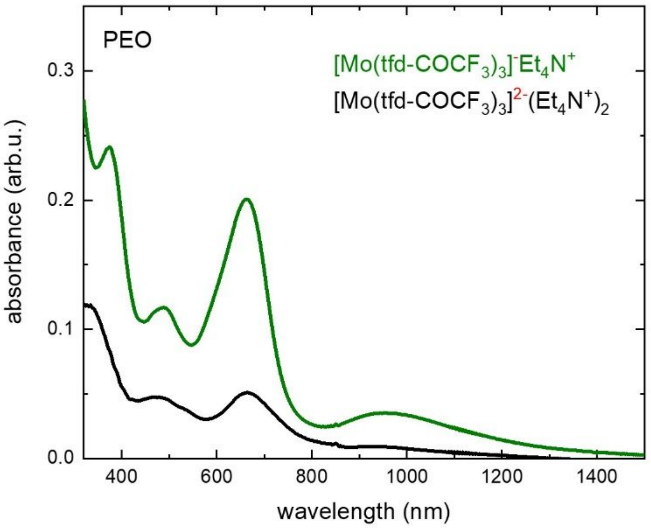

Figure S1. UV-vis spectra of $[Mo(tfd\text{-}COCF_3)_3]^{-}Et_4N^{+}$ and $[Mo(tfd\text{-}COCF_3)_3]^{2-}(Et_4N^{+})_2$ dispersed in a PEO matrix.

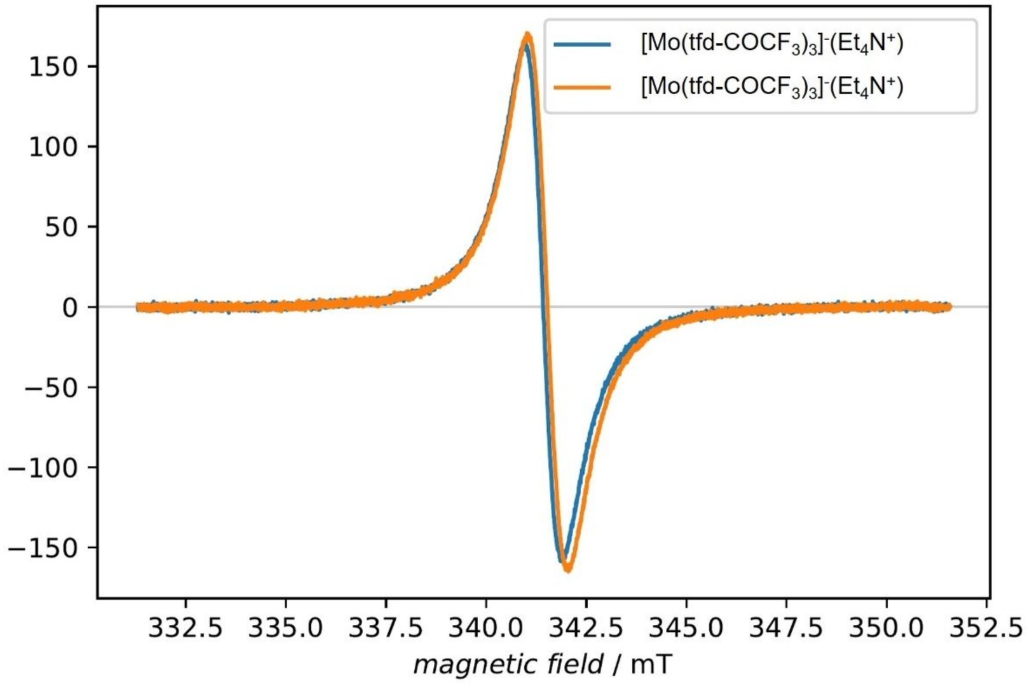

Figure S2. EPR spectra of a thin film of $[Mo(tfd\text{-}COCF_3)_3]^{-}Et_4N^{+}$ dispersed in a PEO matrix recorded at 0° and 90°.

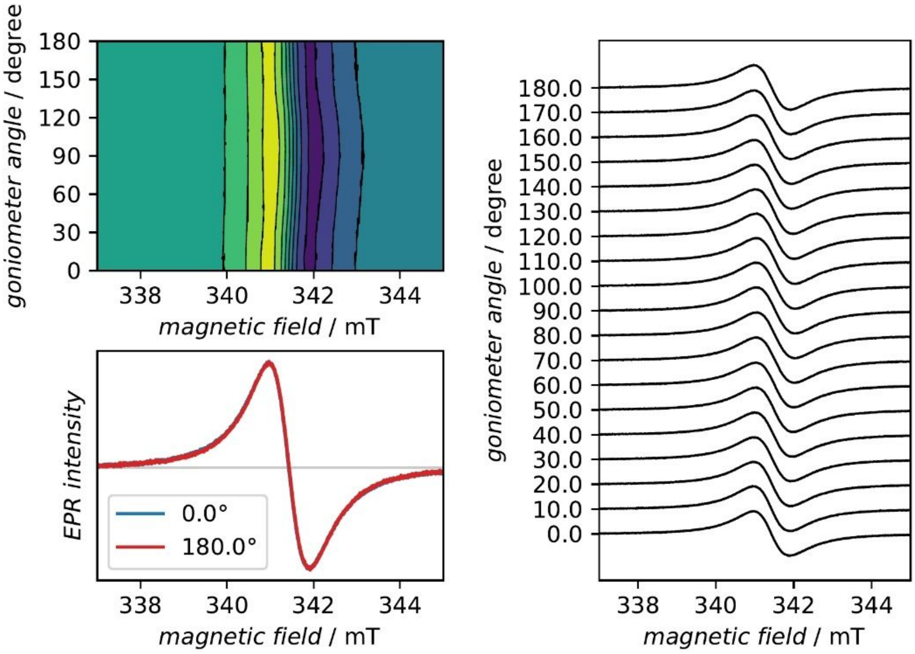

Figure S3. Angular-dependent EPR spectra of a thin film of $[Mo(tfd\text{-}COCF_3)_3]^-Et_4N^+$ dispersed in a PEO matrix. Spectra have been recorded using a motorized goniometer with an accuracy of 0.125°. The sample has been manually positioned within the spectrometer initially with the substrate plane parallel to the magnetic field (with an accuracy of ±5°).

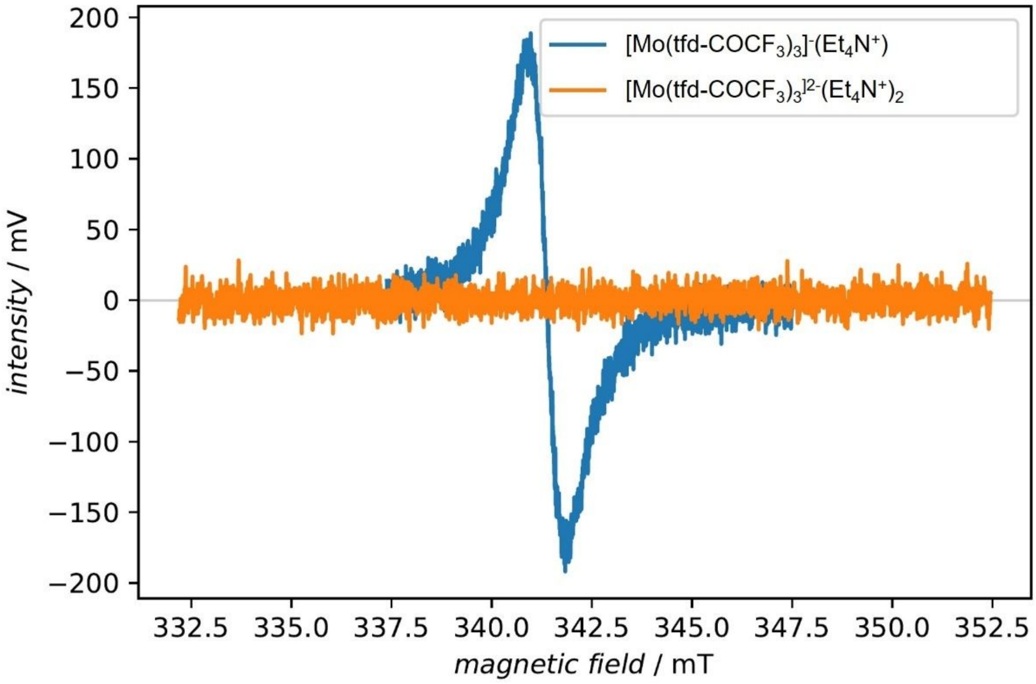

Figure S4. Non-smoothed EPR spectra of $[Mo(tfd\text{-}COCF_3)_3]^-Et_4N^+$ and $[Mo(tfd\text{-}COCF_3)_3]^{2-}(Et_4N^+)_2$ dispersed in a PEO matrix.

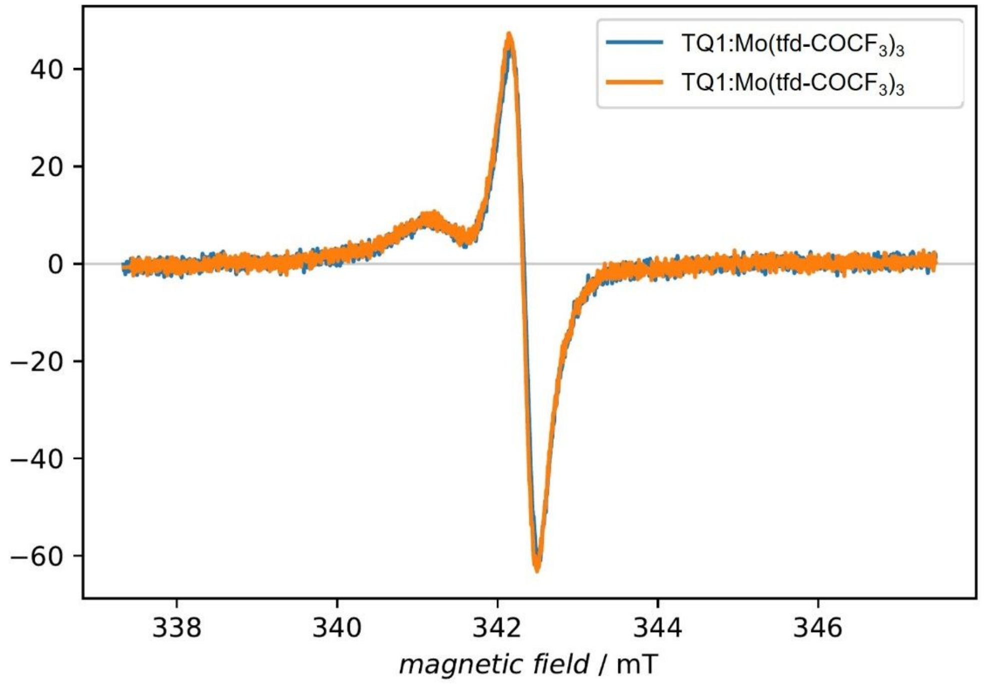

Figure S5. EPR spectra of TQ1 sequentially doped with Mo(tfd-$COCF_3$)$_3$ recorded at 0° and 90°.

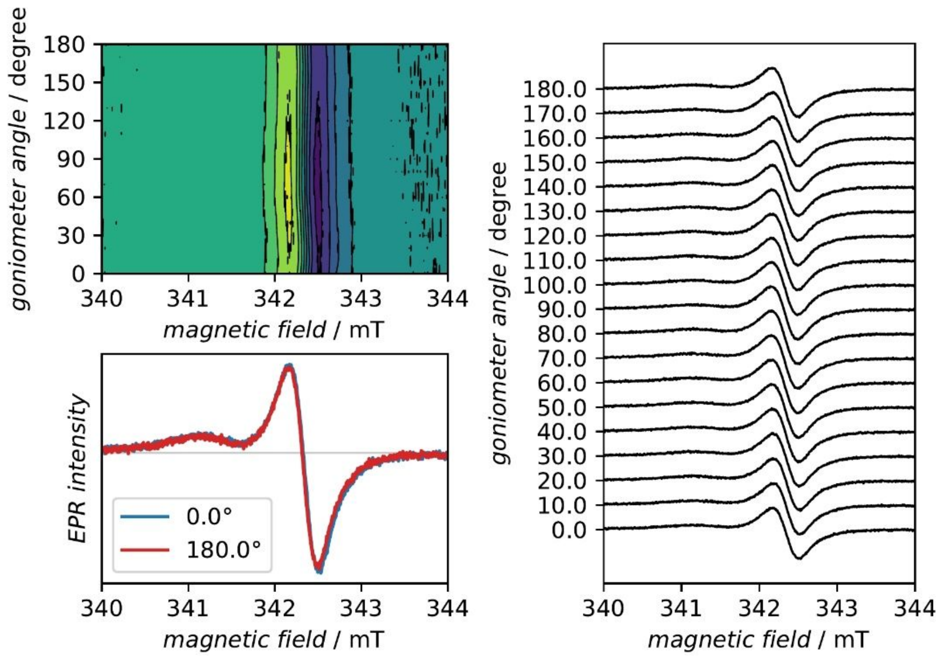

Figure S6. Angular-dependent EPR spectra of a TQ1 sequentially doped with Mo(tfd-$COCF_3$)$_3$. Spectra have been recorded using a motorized goniometer with an accuracy of 0.125°. The sample has been manually positioned within the spectrometer initially with the substrate plane parallel to the magnetic field (with an accuracy of ±5°).

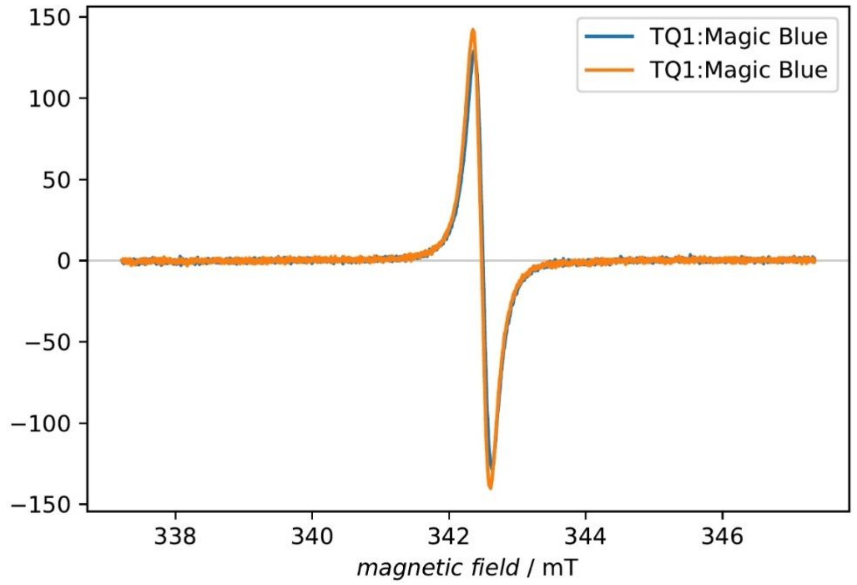

Figure S7. EPR spectra of TQ1 sequentially doped with Magic Blue recorded at 0° and 90°.

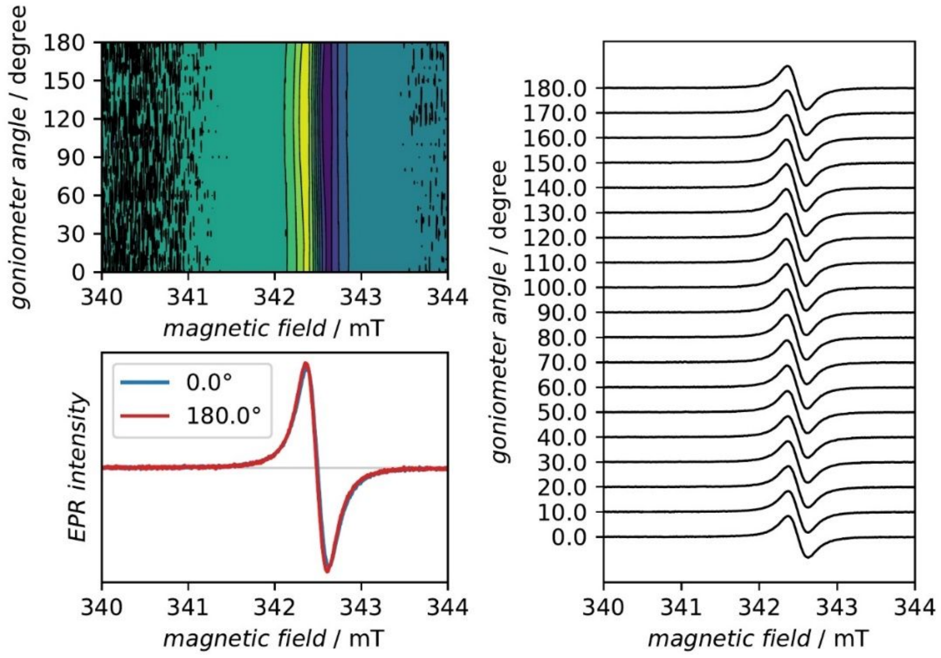

Figure S8. Angular-dependent EPR spectra of a TQ1 sequentially doped with Magic Blue. Spectra have been recorded using a motorized goniometer with an accuracy of 0.125°. The sample has been manually positioned within the spectrometer initially with the substrate plane parallel to the magnetic field (with an accuracy of ±5°).

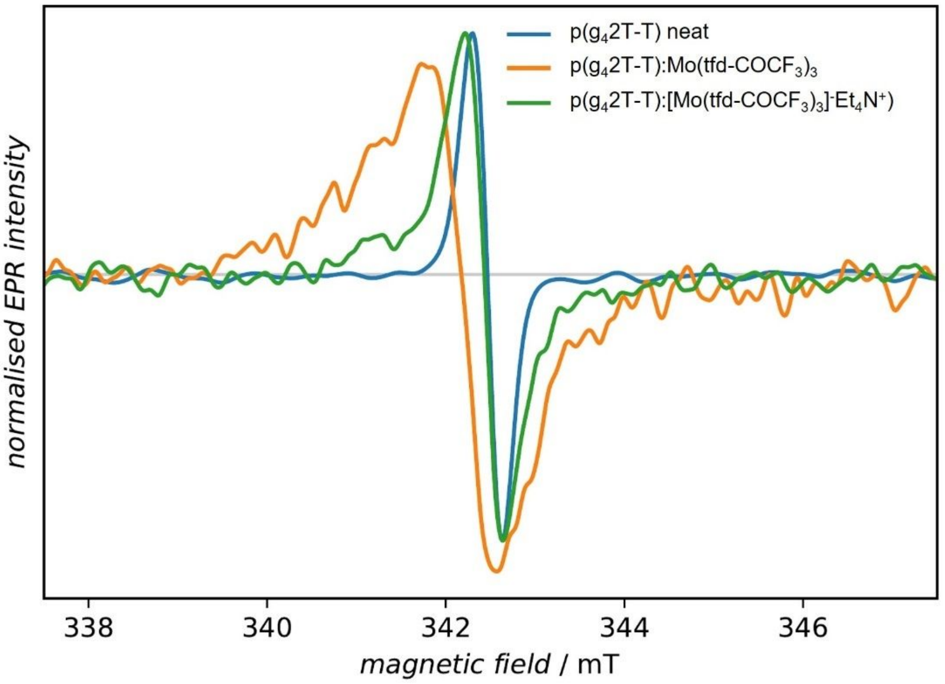

Figure S9. Non-smoothed EPR spectra of neat p($g_4$2T-T) and p($g_4$2T-T) sequentially doped with Mo(tfd-$COCF_3$)$_3$ and [Mo(tfd-$COCF_3$)$_3$]$^-$Et$_4$N$^+$.

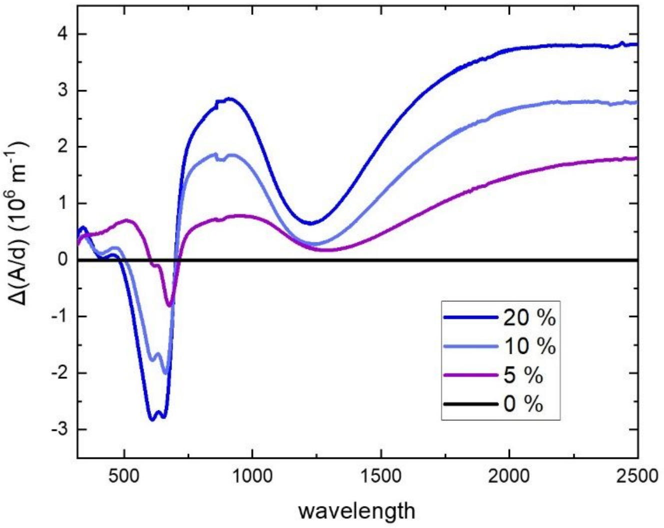

Figure S10. UV−vis−NIR absorbance spectra displaying the difference in thickness-normalized absorbance Δ(A/d) between the spectra of neat p($g_4$2T-T) and p($g_4$2T-T) co-processed with 5 mol%, 10 mol% and 20 mol% of Mo(tfd-$COCF_3$)$_3$.

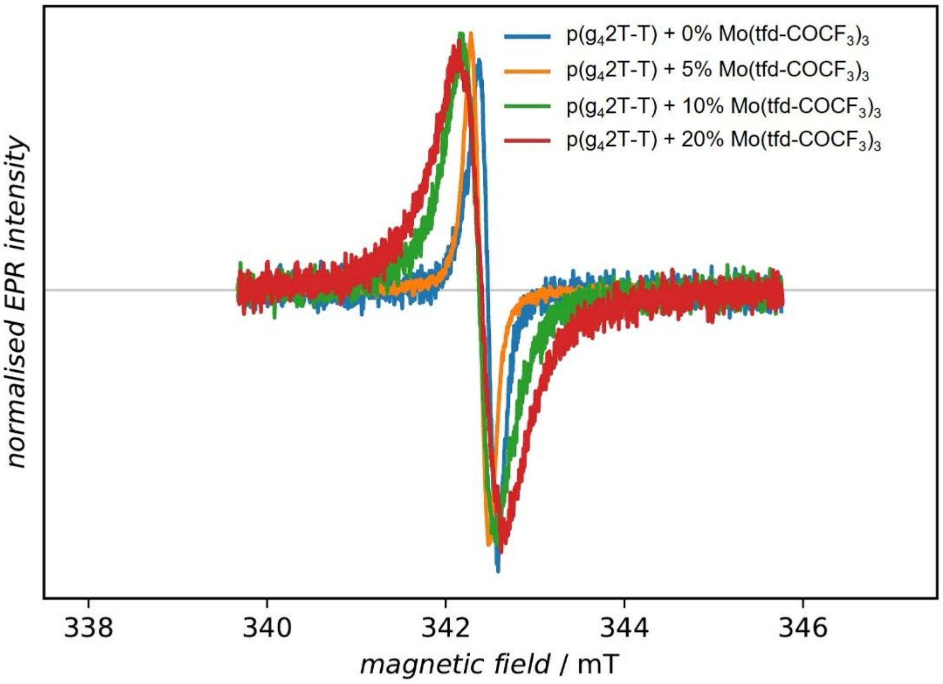

Figure S11. Non-smoothed EPR spectra of co-processed p($g_4$2T-T):Mo(tfd-$COCF_3$)$_3$ films.
